# Why medical students choose orthopedic surgery as a specialty?

**DOI:** 10.11604/pamj.2015.20.364.5807

**Published:** 2015-04-14

**Authors:** Moncef Erraji, Abdessamad Kharraji, Najib Abbasi, Abdeljawad Najib, Hicham Yacoubi

**Affiliations:** 1 Orthopedic Surgery Unit and Traumatology, University Hospital of Oujda, Morocco

**Keywords:** Surgery, training, traumatology-orthopedic surgery, education

## Abstract

Before the crisis announced the Moroccan surgery, the objectives of this study were to analyze the choice of specialties newly appointed to the internal review and the guidance of medical students and to determine the factors influencing this choice. Data on specialty choice students were analyzed and a questionnaire was offered to students of Morocco at the beginning of academic year 2013-20014 The form consisted of questions on the year of study. sex, professional guidelines and reasons for choice. candidates were male, the average age of our residents was 28 years. We also noted the importance of the passage as well as external service trauma. Care provided to patients, lifestyle and income reported by 85% of respondents to be the most important factors to pursue orthopedics as a career. The TR-Orth is now a specialty that responds to a positive choice. The choice of TR-Orth by students at the end of medical school curriculum is reinforced by teaching and practicing the specialty during the internship. The overall training is unsatisfactory overall. Students would deepen in some areas. This study confirms that there is currently a shift in trauma surgery, mostly induced by an a priori negative for particular workloads.

## Introduction

Before any specialization, medical studies are organized in morocco in six years during which we separate a first cycle of medical studies of two years, of the second cycle of four years. In the term of these six years of formation, some students appear at the test classifying of internship others prefer to take the competition of residency what allows them to choose, according to the ranking a specialty this competition is very important as it determines the vocational guidance of the future students. The traumatology - orthopedics is a desirable profession for the graduats in medicine the competition and the number of candidats were superior than posts offered by the ministry of health every year making access to the specialty increasing difficult. The different series of literature underlined the importance of the training orthopedic - traumatology with 45% of medicine students have not received courses of traumatology during their externship passage [1–5]. The objectives of this study were to analyse the choices of specialty of the internals recently appointed to the classifying examination as well as the orientations of the medical students and to determine the factors influencing this choice.

## Methods

We led a medical investigation for the resident's exerting the traumatology as a specialty on the three CHU, Oujda, Fes and Rabat. The criteria of inclusions: the residents of traumatology; foreign residents who cross their training here in Morocco; the internals spend theirs desiratats in traumatology-orthopedic service. The criteria of exclusions: externat; the specialists who spend their equivalence. Meanwhile, a questionnaire was sent by email or distributed personally to the students, all students received explanations on the purposes of the study, its confidential nature and they were free not to answer. The questionnaires were anonymous including 24 questions. The candidates left in two groups. (group1) resident on title and (group 2) resident on competes. The form was made of questions on: general information; the university curriculum; the impact of university training courses and medical exercise; the impact of teaching and the teacher; socio-economic factor. 45 people answered to questionnaires. 29 candidates for group 1 and 16 for the group 2. The results are presented in the form of average medium of quantitative variables (more or less standard deviation) and percentage for the nominal variables.

## Results

**Figure 1:**
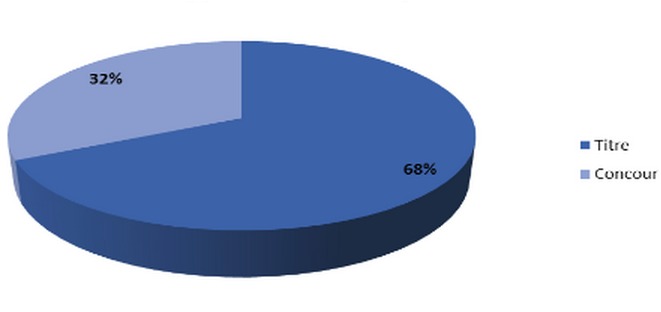
Type of residency

**Figure 2:**
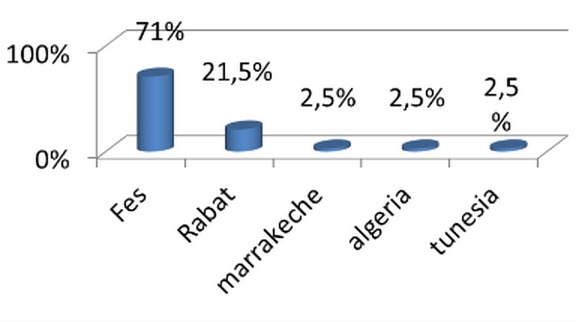
Faculty of general medicine

**Figure 3:**
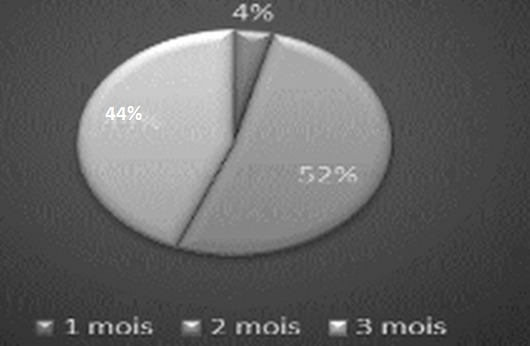
Length of externship stage

**Figure 4:**
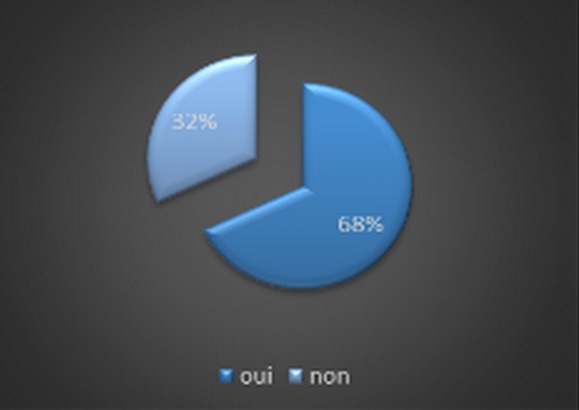
Stage of traumatology

**Figure 5:**
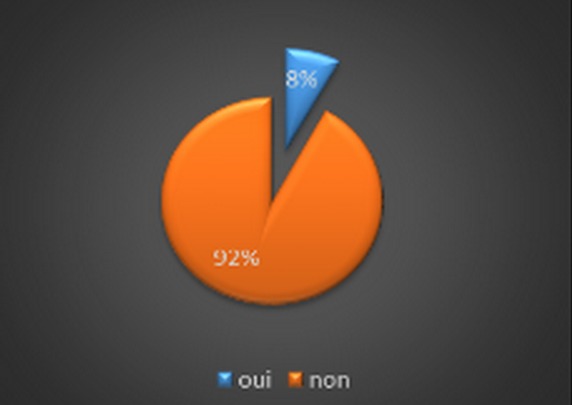
Period of exercise in public/private sector

We led a medical investigation in 3 CHU on 45 residents exercing the specialty of traumatolgy - orthopedics. None of the respondents were excluded for a motive for an incomplete investigation as long as they have answered all the questions consequently a candidate who refused to fill the questionnaire without clear reason. The results were as following: all the candidates were male. The average age of residents was 28 years and 1 month and ranged from (25-33 years). The Candidates are divided on two groups (Figure 1): residents who answered the questionnaire, 56,5% were single and 40,5% married; it was found that almost 71% of residents questioned made their studies of general medicine at the faculty of medicine of Fes (Figure 2). The majority of respondents 87% (n = 39) exercising their specialty in the faculties of Rabat and Fes; it was not that 67%of candidates having benefited from a passage by service of orthopedic surgery during their internship, with almost all had crossed a duration more than two months (Figure 3); we also noted the importance of passing as well as external to the service of traumatology on the definitive decision of the choice of specialty 68% of group 1 we were had on training of TR-OR during the period of internship, on the other hand the majority of the residents on title had no period of exercise as general practitioner in the public or private sector (Figure 4 and Figure 5). This passage as an internal had a big influence to choose a career in orthopedics, for against 24% of questioned doctors think that they had no great interest in the point to decide to do this specialty.

**Figure 6:**
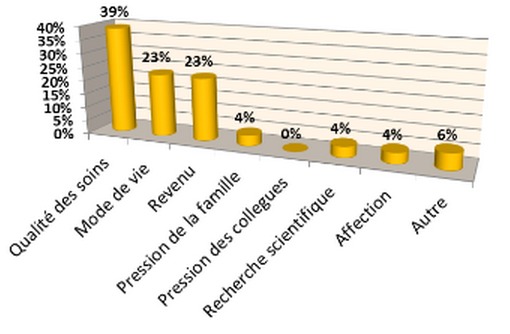
Factors influencing the choice of speciality

Group 2: 75% we had passage to service of TR-OR during their training device, and all respondents from group 2 were asked to estimate the importance of training of traumatology in the choice of the specialty, with most of residents think that training 7_th_year of medicine had a big influence on this choice. Passing through a device and as a general practitioner in group 2 had a great influence on the choice of orthopedic traumatology as a future career placement. Factors career choice: the questions which are asked by 17 to 20 determine the factors influencing the choice of specialty. The care supplied to the patients, lifestyle and income are declared by 85% of respondents to be the most important factors to pursue the orthopedics as a career (Figure 6). The course of the pathology of musculoskeletal system had no big influence in the choice of specialty to 55% of residents questioned. On the other hand almost 73% of the candidates see the ruling atmosphere in their service being a determining and decisive factor. We estimated the importance of income on their choice of the career on a scale of four points, 1 being the most important and 4 being the least important the quoted materiel has not been chosen by five meets as a decisive factor. However, students are asked about individuals who most influenced the choice of residency, almost half answered that the former residents in training, helped in the choice of this domain, on the other hand the skill of professors influenced only 13%; the ranking was important in the choice, the view which 78% of the candidates answered yes, against 12% say no. Finally all the residents were questioned if they are going to do the same choice, with 70% had answered yes for 30% not.

## Discussion

The orthopedic traumatology is besides an extensive surgical specialty requiring multiple skills .the projections on the demography of the traumatologists in the coming years are disturbing. Part of skills of our specialty is already provided by the general practitioner in association with the orthopedic .this collaboration risks to strengthen in the coming years in front of the announced shortage. About half of whom with general practice. It is therefore important that these future general practitioners can benefit from a quality university education in traumatology. There is at present in the scientific literature no study having tried to estimate the satisfaction of the medical students concerning their university education of the traumato-orthopaedics. The education dispensed by the faculties rests essentially on courts of pathologies since decade, what does not correspond to the wish of the questioned. We can make only hypotheses on these results: the organization of the courts being identical between the faculty and the hospitable internships the quality of the supports of court would be better with clinical cases, as well as the quality of the education organized by the faculty remains to improve. These results have to bring us to a certain questioning. It is all the same useful to remind that the university education supplied by the faculties has for only ambition no preparation of the course. The faculty has for mission to train future doctors traumatologist whose skills cannot correspond that in a simple accumulation of knowledge in this connection, the practical training of traumatology seems beneficial to the students questioned within the framework of their formation: seen that 67% in our series had formation in this domain, so putting a major inconvenience for the recruitment of the students in this profession. This result may seem surprising since these formations require an investment of precious time and sometimes contrasts with the poor attendance of some of our students. Bernstein et al [6] showed that only 55% of medical graduates had a compulsory education in musculoskeletal discipline, including orthopedics and rheumatology. The other series in the literature have reported the importance of clinical training for the choice of orthopedics. Maintaining the course currently mandatory trauma also seems necessary and even desired by residents. Our data indicate that a significant proportion of group 1 were influenced by the passage as an intern serving traumatology-orthopedics, the same thing for that was more influenced by passing through a device placement as well as physician group 2 generalist. We sought to better understand the factors that motivated students to pursue an orthopedic career.

The Academy of Surgery recently observed that instead of surgical discipline should always be upgraded in our health care system and in our society. The two key words in this revaluation saving surgery are: hardship and compensation. Remuneration Moroccan surgeons are lower, first, to those of other disciplines and on the other hand, gains exercisers foreign surgeons in countries where the standard of living is comparable to that of Morocco. The motivations of future surgeons were dominated, besides the interest of pathologies, by the possibility of liberal activity, quality of life and income. This is comparable to other global studies. This has already been observed in the United States, where the desire for a “good” quality of life was responsible for more than half of the change of professional direction of future physicians between 1996 and 2002 [7]. The financial aspect is a fairly new data for the French doctors and remains well below the Anglo Saxon countries, in some studies, 40% of American students say they do not want to become general because of insufficient income [8]. Current developments reforms to reduce your self-employment earnings and potential future surgeons are potentially harmful on surgical vocations. It will be difficult to continue to expect motivate surgeons opting out of the utopia of one bonus linked to the vocation [9]. The feminization of the medical population is observed in developed countries [10, 11] and the United States, the student rate increased from 13% in 1970 to 49% in 2007 [12]. This radical change in the medical population is obviously going to lead to changes in the choice of specialties. A study in the United States, found that among 292 women included in the survey only 7% chose orthopedics as a specialty [13]. In our study, men were significantly more attracted to the specialty of orthopedics. The area has not had much success as much as other surgical specialties in recruiting residents’ female sex despite the feminization of the medical school. The woman was able to get a position as a professor of orthopedic traumatology, this brings us, that in a strategy to increase the representation of women in orthopedics to Morocco, we will have a special care of women during their passage into orthopedic services which will encourage them to consider an orthopedic career. There are several limitations in this study, all first we were unable to assess the female candidates who are absent from this survey, we also interviewed and evaluated just too limited sample, it was necessary to carry out a similar study on a broader base of medical school and the need to develop a statistical analysis that could eventually provide a larger sample of women candidates. Because of this limitation on the number of CHU interviewed, we chose to focus on the description of the data obtained in our analysis instead of insisting on statistical significance, and more study was based on a survey asking residents to remember the reasons and factors influencing their career choices.

## Conclusion

The answers to the disaffection of surgery are multiple and often expensive. However, some of them are obvious from reading this study. If Morocco wants to continue to develop trauma surgeons skilled, engaged and volunteers in this discipline, it will probably lead a real incentive policy and provide the means for surgery to be an upgraded profession whose hardship is finally recognized. The stakes are high because it is the price that young people return to surgery.
